# Thiram Determination in Milk Samples by Surface Plasmon Resonance Based on Molecularly Imprinted Polymers and Sulphur-Doped Titanium Dioxide

**DOI:** 10.3390/bios14070329

**Published:** 2024-07-03

**Authors:** Sezen Harmankaya, Hacı Ahmet Deveci, Ahmet Harmankaya, Fatma Hazan Gül, Necip Atar, Mehmet Lütfi Yola

**Affiliations:** 1 Department of Food Processing, Kars Vocational School, Kafkas University, Kars 36000, Turkey; sezen.harmankaya@kafkas.edu.tr; 2 Department of Nutrition and Dietetics, Faculty of Health Sciences, Gaziantep University, Gaziantep 27000, Turkey; h_ahmet_deveci@gantep.edu.tr; 3 Department of Chemistry, Faculty of Science and Literature, Kafkas University, Kars 36000, Turkey; ahmetharmankaya@kafkas.edu.tr; 4 Department of Nutrition and Dietetics, Faculty of Health Sciences, Mersin University, Mersin 33343, Turkey; fatmagul@mersin.edu.tr; 5 Department of Chemical Engineering, Faculty of Engineering, Pamukkale University, Denizli 20160, Turkey; natar@pau.edu.tr; 6 Department of Nutrition and Dietetics, Faculty of Health Sciences, Hasan Kalyoncu University, Gaziantep 27000, Turkey

**Keywords:** thiram, surface plasmon resonance, molecular imprinting, nanocomposite, milk analysis

## Abstract

In this work, a new surface plasmon resonance (SPR) sensor based on sulphur-doped titanium dioxide (S-TiO_2_) nanostructures and molecularly imprinted polymer (MIP) was presented for thiram (THI) determination in milk samples. Firstly, the S-TiO_2_ nanomaterial with a high product yield was prepared by using a facile sol-gel hydrolysis technique with a high product yield. After that, UV polymerization was carried out for the preparation of the THI-imprinted SPR chip based on S-TiO_2_ using a mixture including ethylene glycol dimethacrylate (EGDMA) as the cross-linker, N,N′-azobisisobutyronitrile (AIBN) as the initiator, and methacryloylamidoglutamicacid (MAGA) as the monomer. The reliability of the sensor preparation procedure has been successfully proven by characterization studies of the prepared nanomaterials and SPR chip surfaces through spectroscopic, microscopic, and electrochemical methods. As a result, the prepared SPR sensor showed linearity in the range of 1.0 × 10^−9^–1.0 × 10^−7^ M with a detection limit (LOD) of 3.3 × 10^−10^ M in the real samples, and a sensor technique for THI determination with high sensitivity, repeatability, and selectivity can be included in the literature.

## 1. Introduction

Milk and dairy products are basic foodstuffs for humans. However, they are also risky products as they can contain residues of chemicals such as veterinary drugs, pesticides, mycotoxins, heavy metals, and similar substances [1]. In particular, pesticides, widely used to increase agricultural production and obtain quality products, are among the major chemicals that can form residues in food. When pesticides are applied by spraying, they are partly lost through evaporation and dispersion, while the rest remains on the plant and soil surfaces. Furthermore, the uncontrolled and unintentional use of pesticides can cause significant damage to nature and the environment [2,3]. Depending on the dose and duration of exposure, humans may experience acute and chronic poisoning, as well as carcinogenic, mutagenic, and teratogenic effects [4].

THI is a pesticide with a long history of use in agricultural production. It has been in use for more than 80 years and is effective in controlling fungal diseases in many crops [5]. While THI is crucial in managing plant diseases, its numerous detrimental effects on humans are well-documented. This widely used pesticide can cause a range of adverse effects, including skin diseases, headaches, gastrointestinal problems, and liver damage due to the release of carbon disulfide [5,6]. Therefore, regular residue analysis in milk samples is critical to identify potential risks in advance and protect public health. Given the health problems caused by pesticide residues in foods, there is an increasing need for new methods that provide faster results and are easily applicable.

Currently, TiO_2_ is widely used as an efficient and affordable nanocatalyst for analyzing toxic components in real samples [7,8]. Nonetheless, with a band gap of 3.25 eV, TiO_2_ can only harness around 3–4% of the UV light spectrum for activation in photocatalytic studies [9]. The sensor and photocatalytic efficiency of TiO_2_ can be increased via doping with certain metals and heteroatoms [10]. A decrease in TiO_2_′s band gap can be achieved by using non-metal atoms such as sulphur or nitrogen. The treatment of heteroatom doping on TiO_2_ is an effective method for decreasing band gap values. Sulphur or nitrogen atoms can occupy vacant titanium or oxygen ion sites in the lattice structure. Consequently, the decrease in the band gap occurs by mixing sulphur in its 3p state with the valance band [11]. In the literature, the preparations of TiO_1−x_S_x_ or TiO_1−x_N_x_ nanostructures are achieved via the electronegativity differences between sulphur, nitrogen, and oxygen atoms, providing the shift to the visible light region [12]. In addition, the photocatalytic removal of methylene blue has been performed by using sulphur-doped titanium dioxide nanostructure [13,14]. In a photocatalytic removal study, the sulphur atom, acting as an anion, replaced the oxygen lattice in the TiO_2_ nanostructure. In another study, the incorporation of sulphur atoms resulted in the replacement of Ti ions in the TiO_2_ nanostructure [15]. Because there are several types of sulfur sources, it has been shown that sulfur atoms can exist in multiple oxidation states, such as S^2−^, S^4+^, or S^6+^ [16].

Molecularly imprinted polymers (MIPs) are specific and selective polymers for creating an ‘artificial lock’ that fits the ‘key-lock’ model, mostly similar to enzyme-substrate or antigen-antibody interactions. Obtaining the desired regular, specific, and selective regions in artificial polymers during the imprinting process forms the basis of molecular recognition [17]. MIPs have been proven to be effective in selectively adsorbing and detecting numerous target analytes, functioning as synthetic antibodies with specific recognition capabilities for the analyte. Compared to natural antibodies, MIPs provide numerous benefits, including high selectivity, straightforward synthesis, affordability, and chemical stability [18]. To obtain MIPs, three primary steps are involved: the chemical bonding or physical interaction between the template molecule and the functional monomer; the polymerization process initiated in the presence of an initiator, porogen, and cross-linker; and finally, the extraction of the template molecule [19].

SPR is a physical phenomenon that is based on the combination of a light photon with the electrons of atoms on the surface covered with a thin, nanosized metal or occurs through the energy transfer between light photons and metal electrons. This technique is based on the plasmonic fluctuations that occur on metal surfaces as a result of the absorption of a laser beam. As a result of this absorption, certain changes occur in the band gap of the sensor material. The changes in the band gap affect the refractive index and cause changes in the SPR determination. Even the small band gap increases the intensity of the SPR peak [20]. Because of this, the S-TiO_2_ material, which has a relatively smaller energy band gap than the undoped TiO_2_ material, was synthesized in this study, and more sensitive results were obtained in SPR sensor applications.

This paper exhibited a new SPR method for thiram analysis based on sulphur-doped titanium dioxide nanostructures and molecularly imprinted polymers. Firstly, after synthesizing S-TiO_2_ nanomaterial using a sol-gel hydrolysis technique, a THI-imprinted SPR sensor based on S-TiO_2_ was designed using UV polymerization in the presence of THI and MAGA monomer. The recovery application in milk samples was successfully implemented, achieving a high recovery rate. Hence, this THI-imprinted SPR sensor based on S-TiO_2_, which was developed for food safety and access to healthy foods, can provide a new perspective on healthy living in terms of safe food consumption [21,22]. 

## 2. Materials and Methods

### 2.1. Chemicals and Apparatus

THI, ziram (ZIR), thiophanate (THP), ferbam (FER), disulfiram (DIS), titanium (IV) isopropoxide (TTIP), thiourea, MAGA, EGDMA, 2-hydroxyethylmethacrylate (HEMA), AIBN, phosphate buffer (PBS), and sodium chloride (NaCl) were purchased by Sigma-Aldrich Merck Group company (St. Louis, MO, USA).

Transmission electron microscopy (TEM, JEOL 2100 TEM, Tokyo, Japan), a PHI 5000 Versa Probe-type X-ray photoelectron spectroscope (Tokyo, Japan/New York, NY, USA), a Bruker-Tensor Fourier transform infrared spectrometer (FTIR, Tokyo, Japan), and X-ray diffraction (XRD, Rikagu Miniflex X-ray diffractometer, Tokyo, Japan) were employed to characterize the nanostructures of undoped TiO_2_ and S-TiO_2_ nanomaterials.

Tapping mode AFM was utilized (Nano Magnetics Instruments, Oxford, UK). SPR chips were installed on a 2 μm × 2 μm sample holder with a 128 × 128 pixel resolution. Measurements belonging to six different areas were taken with a scan rate of 2 μm·s^−1^ in an atmosphere of air.

The GenOptics SPR system (Calgary, AB, Canada) was utilized for analytical applications. Finally, the Gamry Reference 600 workstation (USA) was used for the electrochemical investigations via electrochemical impedance spectroscopy (EIS) and CV.

### 2.2. Preparation of S-TiO_2_

The sol-gel hydrolysis of TTIP was carried out for the preparation of the TiO_2_ nanocatalyst. TTIP solution (2.0 mg mL^−1^) in ethanol was distilled using an ultra-pure water/ethanol mixture (10.0 mL, 1:1 *v*/*v*) under strong stirring conditions. Then, the prepared gel was transferred into a Teflon autoclave and heated at 70 °C for 36 h. The resulting product (undoped TiO_2_) was dried at 70 °C. The sol-gel hydrolysis procedure described above was repeated with the addition of thiourea and TTIP solution (2.0 mg mL^−1^) in ethanol to complete the production of S-TiO_2_. The sulphur dopant was equivalent to 0.1 atomic percent and was introduced by adding the appropriate amount of thiourea. The sample was tagged as S-TiO_2_ (0.1 at% sulphur on TiO_2_). The amount of sulphur was kept low to avoid agglomeration in harmony with the literature during the doping process and to obtain the most efficient SPR signals [9].

### 2.3. SPR Chip Modification Using S-TiO_2_ and the Development of the THI-Imprinted S-TiO_2_/SPR Chip

First, the supplied SPR chips were cleaned in a shaking bath system for 30 min with acidic piranha solution containing (3:1) H_2_SO_4_/H_2_O_2_ (25.0 mL *v*/*v*). After 30 min, SPR chips were dried at 25 °C under nitrogen gas conditions and were made ready for use. After the prepared S-TiO_2_ solution (5.0 mg mL^−1^) was dropped onto the cleaned SPR chip surface, the S-TiO_2_/SPR surface was prepared with the help of gold-sulfur bonding [23]. After preparing the MAGA-THI complex at a 2:1 molar ratio with the addition of PBS (1.0 mL, pH 6.0) for 30 min, the AIBN (2.5 mg), HEMA (0.5 mL), and EGDMA (1.0 mL) mixtures were slowly transferred into the MAGA-THI complex solution (0.5 mL). Nitrogen gas was used to remove impurities from this final solution, taking approximately 15 min. A homogeneous, monolayer polymerization solution was prepared on the S-TiO_2_/SPR surface by dropping the prepared dispersion (50.0 µL) using the spin coating method onto the SPR chip surface for 15 min. After UV polymerization by UV light for 15 min, a THI-imprinted SPR chip was developed (MIP/S-TiO_2_/SPR). In the same way, the non-THI-imprinted SPR chip was developed without the THI molecule (NIP/S-TiO_2_/SPR) using the same procedure described above.

### 2.4. THI Removal from MIP/S-TiO_2_/SPR and the Analysis Process

A total of 1.0 M NaCl (15.0 mL) was used as the desorption agent to eliminate the electrostatic/hydrogen bond interactions between the MAGA monomer and the THI analyte molecule and to create nanocavities specific to the THI molecule. For this purpose, the THI-imprinted SPR chip was kept in a conical flask containing 1.0 M NaCl (15.0 mL) for 1 min. After 1 min, the SPR chip was dried at 25 °C under nitrogen gas conditions.

After the THI-removed SPR chip was placed in the SPR cell, it was placed in PBS solution (2.0 mL, pH 6.0) between 0–10 min with a 1.0 mL min^−1^ flow rate to equilibrate. Afterward, THI adsorption solutions (4.0 mL), each in different concentrations, interacted with the MIP SPR chip for 10 to 50 min with a 1.0 mL min^−1^ flow rate until a constant resonance frequency was reached. After the desorption process was completed by utilizing 1.0 M NaCl solution (2.0 mL) for between 50 to 51 min, the regeneration process was carried out using PBS solution (2.0 mL, pH 6.0), taking 51 to 60 min.

### 2.5. Sample Preparation

The milk samples were prepared for analysis according to our previous study. In this protocol, a milk sample (10.0 mL) was treated with trichloroacetic acid (2.0 mL, 10.0% *m*/*v*) under strong stirring conditions for 30 s and then centrifuged at 5000 rpm for 10 min. Then, the supernatant was diluted with 0.1 M PBS (pH 6.0) for SPR sensor analysis [24].

## 3. Results and Discussion

### 3.1. Characterization of S-TiO_2_

**Figure 1:**
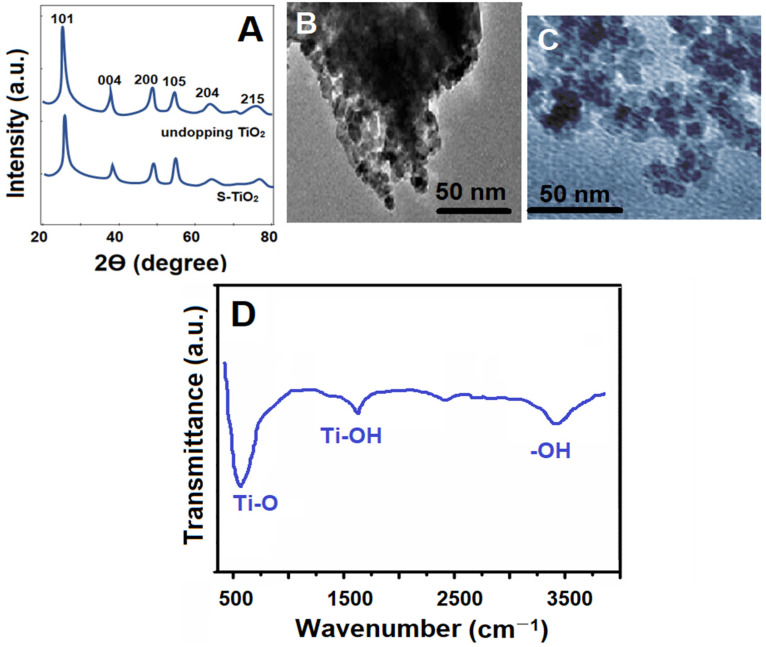
(**A**) XRD patterns of S-TiO_2_ and undoped TiO_2_ nanomaterials; TEM images of (**B**) undoped TiO_2_ and (**C**) S-TiO_2_ nanomaterials; and (**D**) a FTIR spectrum of undoped TiO_2_.

XRD patterns were first recorded for S-TiO_2_ and undoped TiO_2_ (Figure 1A). XRD peaks at 25.31°, 38.14°, 48.07°, 54.09°, and 62.51° corresponded to the (101), (004), (200), (105), and (215) planes for S-TiO_2_ and undoped TiO_2_ nanomaterials. In addition, the crystallite size of S-TiO_2_ nanomaterial was calculated as 5.1 nm using the Scherrer equation [9]. These results proved that S-TiO_2_ and undoped TiO_2_ nanomaterials had similar peak patterns. As shown in the TEM images (Figure 1B,C), spherical particles sized 5.2–9.7 nm were seen. More large particle structures were seen in S-TiO_2_ compared to in the undoped TiO_2_ structure, indicating the successful synthesis of the S-TiO_2_ nanomaterial [9,25]. In addition, Figure 1D shows the FTIR spectra as well as the Ti-O bending mode and the Ti-OH stretching mode at 485 cm^–1^ and 1624 cm^–1^, respectively. Asymmetrical and symmetrical stretches belonging to the −OH group were observed at 3405 cm^–1^. The Ti-OH stretching mode at 1624 cm^–1^ corresponds to the adsorbed H_2_O on the TiO_2_ surface.

XPS was performed on the S-TiO_2_ nanomaterial (Figure S1). The XPS peak at 166.85 eV revealed an S^4+^ ion presence. In addition, XPS peaks at 167.94 eV and 169.12 eV confirmed the presence of S^6+^ ions [15]. Moreover, the observed XPS peaks at 460.17 eV (Ti 2p3/2), 464.08 eV (Ti 2p1/2), 530.78 eV (O1s), and 285.17 eV (C1s) verified the successful synthesis of the S-TiO_2_ nanomaterial [9].

The nitrogen adsorption curve (Figure S2A) was obtained for S-TiO_2_ and undoped TiO_2_ nanomaterials. S-TiO_2_ and undoped TiO_2_ nanomaterials exhibited a type-IV adsorption isotherm typical of H3-type hysteresis. Slit pores formed due to the aggregation of S-TiO_2_ and undoped TiO_2_ nanomaterials. The values of the surface areas of S-TiO_2_ and undoped TiO_2_ nanomaterials were calculated to be 285.78 m^2^ g^−1^ and 224.37 m^2^ g^−1^, respectively, revealing a higher mesopores number in the S-TiO_2_ nanomaterial.

Figure S2B shows the diffuse reflectance spectra of S-TiO_2_ and the undoped TiO_2_ nanomaterials. The undoped TiO_2_ nanomaterial had an absorption peak at about 381 nm and S-TiO_2_ nanomaterial had an absorption peak at about 590 nm [26,27]. In addition, the band gap values of S-TiO_2_ and the undoped TiO_2_ nanomaterial were 1.98 and 3.17 eV, respectively. Hybrid states near the conduction band caused a narrowed band gap in S-TiO_2_; this was due to the substitution of titanium ions in the S-TiO_2_ nanomaterial.

### 3.2. FTIR and AFM Characterizations of THI-Imprinted Film on S-TiO_2_/SPR Chips

**Figure 2:**
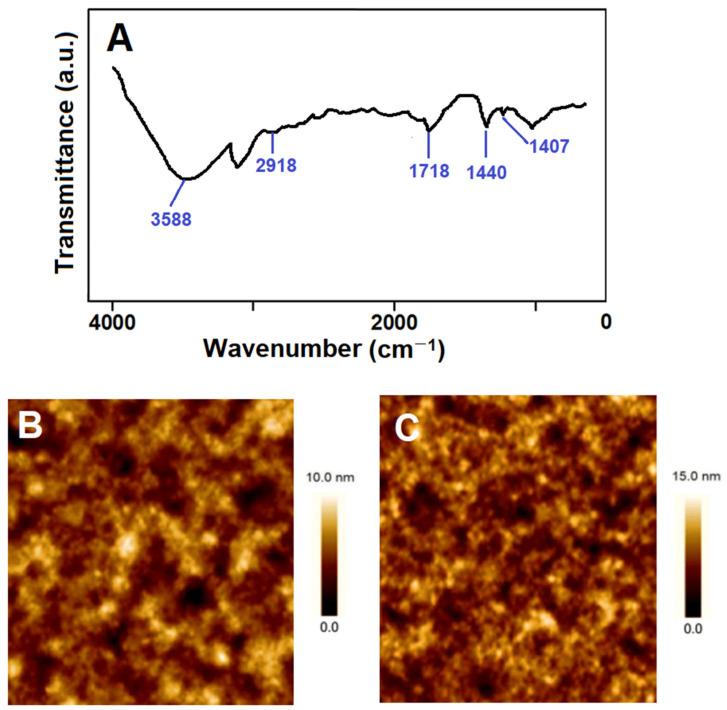
(**A**) FTIR spectra of the THI-imprinted film on the S-TiO_2_/SPR chip; AFM images of (**B**) the bare SPR chip and (**C**) the THI-imprinted film on the S-TiO_2_/SPR chip.

Figure 2A shows the FTIR spectra of the prepared THI-imprinted SPR chip with HEMA and MAGA. Before the removal of THI from the SPR surface, FTIR peaks at –OH were seen at 3588 cm^−1^; the –CH stretching of MAGA was seen at 2918 cm^−1^; –NH bonding corresponding to the amide vibration of MAGA was observed at 1440 cm^−1^; carboxyl-carbonyl stretching was seen at 1718 cm^−1^; and –COO– stretching was observed at 1407 cm^−1^, as shown in Figure 2A [21,22,28]. Thus, the resultant FTIR peaks confirmed the successful imprinting of THI on the S-TiO_2_/SPR chip. Figure 2B,C show the AFM images of the bare SPR chip and the THI-imprinted film on the S-TiO_2_/SPR chip, respectively, and the surface thicknesses were calculated as 2.37 ± 0.07 and 24.31 ± 0.03 nm, respectively, confirming the formation of THI-imprinted polymers on the SPR chip. 

### 3.3. Electrochemical Characterizations of Modified Electrodes with S-TiO_2_ and Undoped TiO_2_ Nanomaterials

Generally, EIS and CV techniques are the electrochemical methods most commonly used to examine the electron transfer phenomenon at the electrode-solution interface and the conductivity properties of the prepared electrode materials (undoped TiO_2_/GCE and S-TiO_2_/GCE). First of all, the observed anodic and cathodic peaks (curve A in Figure S3A) at +0.300 V and +0.600 V on bare GCE were much clearer and the differences between peak potentials decreased when the undoped TiO_2_/GCE was used, owing to the TiO_2_ nanoparticles’ conductive interfaces and applications as catalyists (curve B of Figure S3A) [29,30]. Finally, the highest peak currents and sensor effects were obtained when S-TiO_2_/GCE was used (curve C of Figure S3A). This was because sulphur doping, which caused rapid electron transfer, reduced the conduction band of TiO_2_ [31]. Moreover, EIS graphs were used to prove CV results. The charge transfer resistance (Rct) values were 40 ohms for bare GCE (curve A of Figure S3B), 30 ohms for undoped TiO_2_/GCE (curve B of Figure S3B), and 22 ohms for S-TiO_2_/GCE (curve C of Figure S3B). Thus, these EIS results verify the broad usage possibilities of the S-TiO_2_ nanomaterial in sensor applications.

### 3.4. pH Effect on THI-Imprinted SPR Chips

**Figure 3:**
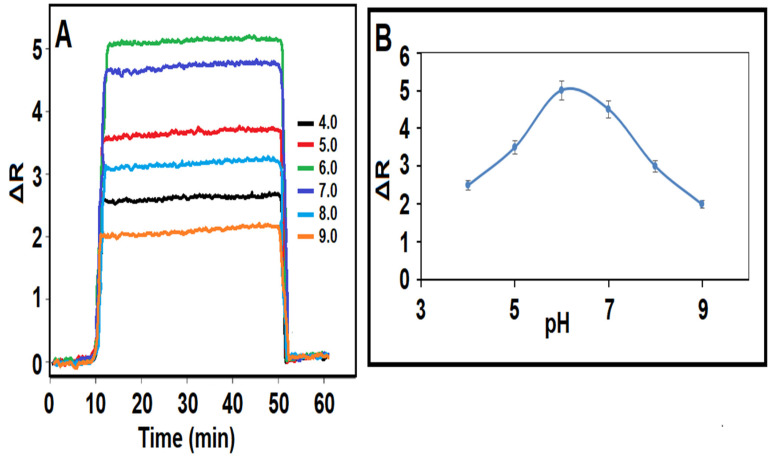
(**A**) SPR sensorgrams for the 10.0 nM THI at different pHs of PBS and (**B**) the effect of pH on THI-imprinted S-TiO_2_/SPR chips (equilibration process between 0 and 10 min; adsorption process between 10 and 50 min; desorption process between 50 and 51 min; regeneration process between 51 and 60 min).

The pH of the working buffer solution is the primary factor influencing the stability of sensor signals obtained in SPR sensor applications. The monomer employed in this investigation, MAGA, has two pKa values (pKa1: 2.10 and pKa2: 4.07). Especially at low pH values, the carboxylic acid groups of the MAGA monomer are present in the anionic phase, and in this case, the electrostatic interactions between the target molecule (THI) and the monomer were many. On the other hand, because the anionic phase state of the THI molecule formed at high pH values, the analyte-monomer bond began to decrease, and in this case, the sensor signals began to decrease accordingly. Thus, pH 6.0 was chosen as the optimum pH value for future analytical applications (Figure 3A,B) [32].

### 3.5. Linearity Range of MIP/S-TiO_2_/SPR Chips

**Figure 4:**
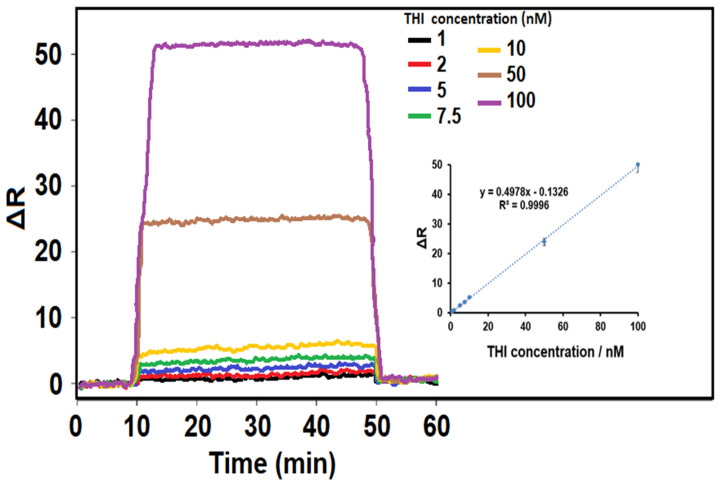
Effect of THI concentration on THI-imprinted SPR chips. Inset: calibration curve of THI concentrations from THI-imprinted SPR chip with a pH 6.0 in PBS (from 1.0 nM to 100.0 nM THI) (equilibration process between 0 and 10 min; adsorption process between 10 and 50 min; desorption process between 50 and 51 min; regeneration process between 51 and 60 min).

SPR is the refractive index change that occurs when two distinct mediums come together. Today, the SPR technique is widely utilized since it can take measurements in real time with great precision and does not require any marking processes. This technique works by sending a laser beam to a metal surface, some of which is then reflected and part of which is absorbed by the metal surface [33]. The primary benefit of SPR-based sensors is their exceptional wavelength sensitivity [34,35]. In this study, SPR signals were linear within the range of 1.0 to 100.0 nM THI (R^2^ = 0.9996), and an calibration equation of y (ΔR) = 0.4978x (C_THI_, nM) − 0.1326 was obtained, as shown in Figure 4. The limits of the quantification (LOQ) and LOD values were 1.0 × 10^−9^ M and 3.3 × 10^−10^ M, respectively (see the Supplementary Materials for the equations). Thus, it is possible to say that a sensor technique with the highest sensitivity, when compared to other THI analysis studies in the literature, is presented here (Table 1). In addition, since the sol-gel hydrolysis technique was used during the sensor preparation, an environmentally friendly material synthesis was achieved with minimal waste generation. Thus, using this sensor, it was shown that it is possible to analyze pesticides found in frequently consumed dairy products quickly and reliably, and an important sensor technique has been developed for food safety.

### 3.6. Recovery

To prove the validity of the prepared sensor, recovery values (%) were calculated by using it on real milk samples. Good recovery values prove that the developed sensor operates with high selectivity and accuracy in real samples. Thus, using the sensor developed in this study, the analysis of pesticides such as thiram in food samples can be carried out with high accuracy and selectivity, thus ensuring the consumption of safe foods. For this purpose, the milk samples prepared for analysis were first divided into four equal parts. Except for the first part, increasing concentrations (2.00, 4.00, and 6.00 nM) of THI standard solution were added to the other three parts. These four real samples were analyzed with the prepared sensor via THI analysis, and the recovery values were calculated. Recovery values of close to 100.0% prove that the prepared sensor can be used with high reliability (Table S1).

### 3.7. Selectivity, Repeatability, and Reusability of MIP/S-TiO_2_/SPR Chips

**Figure 5:**
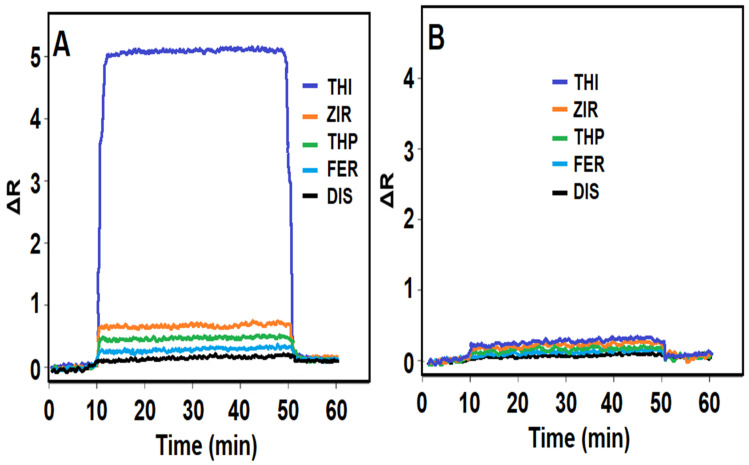
Selectivity tests: SPR sensorgrams of (**A**) MIP/S-TiO_2_/SPR chips and (**B**) NIP/S-TiO_2_/SPR chips with 10.0 nM THI, 1000.0 nM ZIR, 1000.0 nM THP, 1000.0 nM FER, and 1000.0 nM DIS (equilibration process between 0 and 10 min; adsorption process between 10 and 50 min; desorption process between 50 and 51 min; regeneration process between 51 and 60 min).

Figure 5A,B are SPR sensorgrams showing the selectivity of the prepared MIP- and NIP-based SPR sensors in combination with other agents (1000.0 nM ZIR, 1000.0 nM THP, 1000.0 nM FER, and 1000.0 nM DIS). As expected, it was observed that the prepared THI-imprinted SPR sensor showed high selectivity towards THI in combination with other agents. In addition, it was observed that according to k and k’ values, the molecular imprinting process resulted in high selectivity (Table S2).

**Figure 6:**
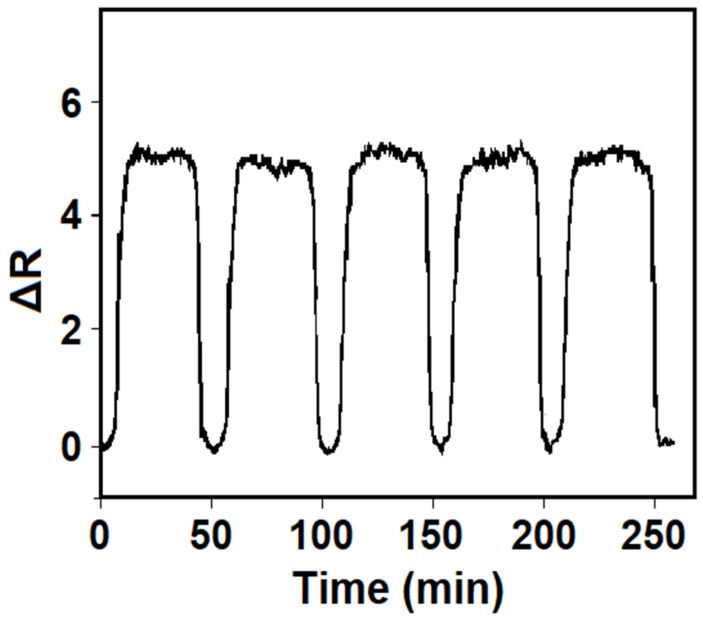
Repeatability of the MIP/S-TiO_2_/SPR chip in 10.0 nM THI.

The SPR sensor prepared for the repeatability test completed five consecutive testing cycles in combination with 10.0 nM THI, and the relative standard deviation (RSD) of the observed SPR signals was measured as 0.19%, demonstrating high repeatability (Figure 6).

Finally, the reusability of the MIP/S-TiO_2_/SPR chip was investigated through 50 consecutive usages of one sensor in combination with 10.0 nM THI, and a 0.87% RSD for the 50 observed SPR signals was calculated. This value indicates the high reusability of the prepared sensor.

## 4. Conclusions

This work reported the development and application of a surface plasmon resonance sensor based on molecularly imprinted polymers and sulfur-doped titanium dioxide on milk samples. The sulfur-doped titanium dioxide nanomaterial had a significant positive impact on SPR sensor response such as reusability and repeatability. Moreover, the developed SPR sensor showed high selectivity and sensitivity in milk samples. For example, a linearity was in the 1.0 × 10^−9^–1.0 × 10^−7^ M range, and an LOD of 3.3 × 10^−10^ M was obtained, thus demonstrating an ultra-sensitive sensor design towards THI pesticide. In conclusion, thanks to this SPR sensor, an important pesticide analysis technique has been developed for food safety.

## Figures and Tables

**Table 1:** The comparison of the MIP/S-TiO_2_/SPR chip’s performance using the reported methods.

Method	Linear Range (M)	LOD (M)	Ref.
Optical fiber probe based on AuNP	1.0 × 10^−7^–10.0 × 10^−4^	5.0 × 10^−10^	[36]
Ratiometric electrochemical method	1.0 × 10^−8^–3.0 × 10^−6^	1.5 × 10^−10^	[37]
AgNPs/CH/office paper	1.0 × 10^−5^–1.0 × 10^−8^	1.0 × 10^−7^	[38]
AuNPs@ZnCo-MOF SERS	1.0 × 10^−7^–1.0 × 10^−4^	1.0 × 10^−7^	[39]
MXene/AgNs SERS	1.0 × 10^−2^–1.0 × 10^−8^	2.1 × 10^−8^	[40]
Fluorescence-DNA-AgNCs	1.2 × 10^−8^–2.0 × 10^−7^	1.0 × 10^−8^	[41]
MIP/S-TiO_2_/SPR chip	1.0 × 10^−9^–1.0 × 10^−7^	3.3 × 10^−10^	This study

## Data Availability

The original data in this study are included in this study, and further inquiries can be directed to the corresponding author.

## Supplementary Materials

The following supporting information can be downloaded at: https://www.mdpi.com/article/10.3390/bios14070329/s1. Figure S1: Surveyed XPS spectra of the S-TiO_2_ nanomaterial. Inset: high-resolution spectra for S2p; Figure S2: (A) Nitrogen-adsorbed isotherms of S-TiO_2_ and undoped TiO_2_ nanomaterials and (B) the diffuse reflectance spectra of S-TiO_2_ and undoped TiO_2_ nanomaterials; Figure S3: (A) CV curves and (B) EIS responses of (a) bare GCE, (b) undoped TiO_2_/GCE, and (c) S-TiO_2_/GCE (redox probe: 5.0 mM [Fe(CN)_6_]^3−/4−^ containing 0.1 M KCl, and potential scan rate of 100 mV s^−1^); Table S1: Recovery results of THI (n = 6); Table S2: k and k′ values of THI-imprinted SPR chips (MIP/S-TiO_2_/SPR chip and NIP/S-TiO_2_/SPR chip) (n = 6).
