# Supplementary material for: Thiram Determination in Milk Samples by Surface Plasmon Resonance Based on Molecularly Imprinted Polymers and Sulphur-Doped Titanium Dioxide
Source: Biosensors (Basel). 2024 Jul 3;14(7):329. doi: 10.3390/bios14070329 (PMC11275002; doi:10.3390/bios14070329)
Supplement: Supplementary file 1 [file biosensors-14-00329-s001.zip › biosensors-3064668-supplementary.pdf]

Supplementary Data

For

Thiram Determination in Milk Samples by Surface Plasmon Resonance Based on Molecularly Imprinted Polymers and Sulphur-Doped Titanium Dioxide

Sezen Harmankaya [1], Hacı Ahmet Deveci [2], Ahmet Harmankaya [3], Fatma Hazan Gül [4], Necip Atar [5] and Mehmet Lütfi Yola [6,*]

[1] Department of Food Processing, Kars Vocational School, Kafkas University, Kars 36000, Turkey; sezen.harmankaya@kafkas.edu.tr
[2] Department of Nutrition and Dietetics, Faculty of Health Sciences, Gaziantep University, Gaziantep 27000, Turkey; h_ahmet_deveci@gantep.edu.tr
[3] Department of Chemistry, Faculty of Science and Literature, Kafkas University, Kars 36000, Turkey; ahmetharmankaya@kafkas.edu.tr
[4] Department of Nutrition and Dietetics, Faculty of Health Sciences, Mersin University, Mersin 33343, Turkey; fatmagul@mersin.edu.tr
[5] Department of Chemical Engineering, Faculty of Engineering, Pamukkale University, Denizli 20160, Turkey; natar@pau.edu.tr
[6] Department of Nutrition and Dietetics, Faculty of Health Sciences, Hasan Kalyoncu University, Gaziantep 27000, Turkey
* Correspondence: mlutfi.yola@hku.edu.tr; Tel.: +90-3422118080; Fax: +90-3422118081

Linearity range

$$LOQ = 10.0\ S / m$$

$$LOD = 3.3\ S / m$$

S: Standard deviation of the intercept and m: Slope of the regression line

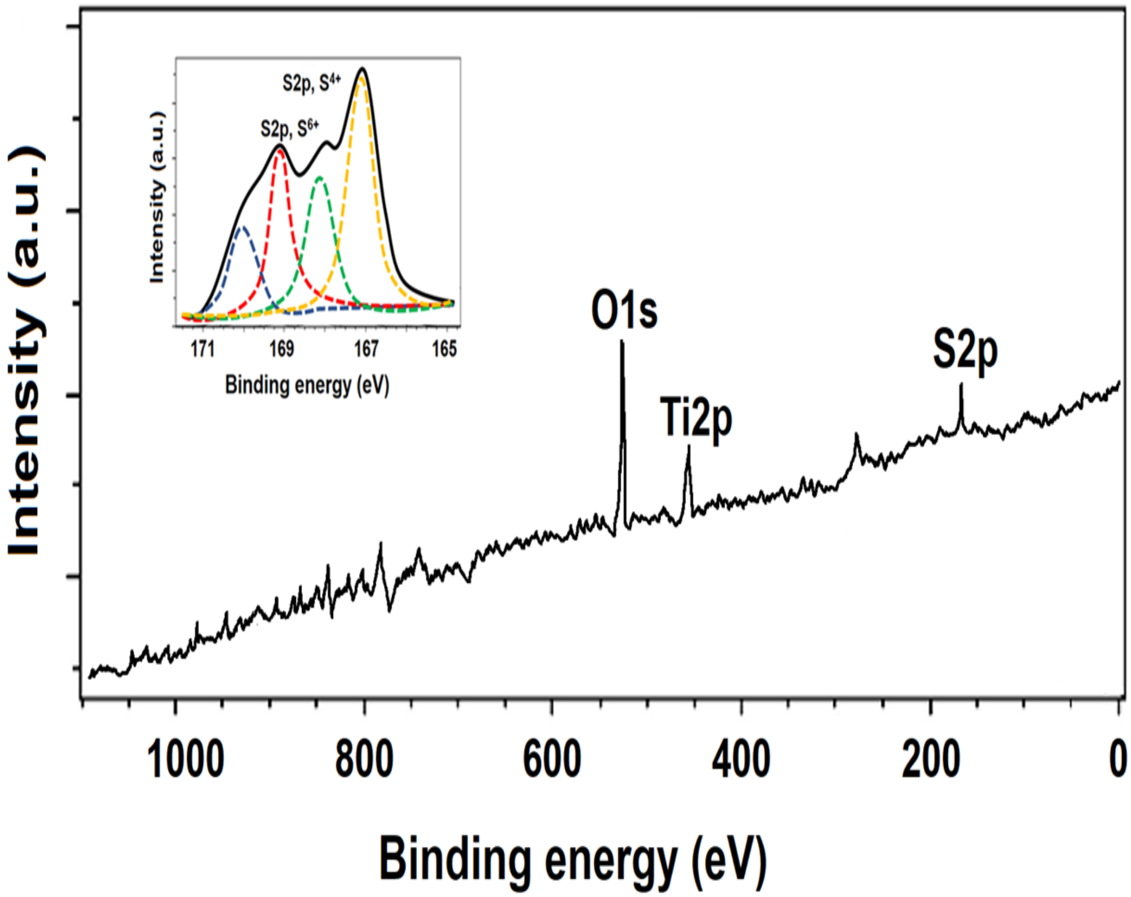

Figure S1. Survey XPS spectra of S-TiO_2 nanomaterial. Inset: High-resolution spectra for S2p

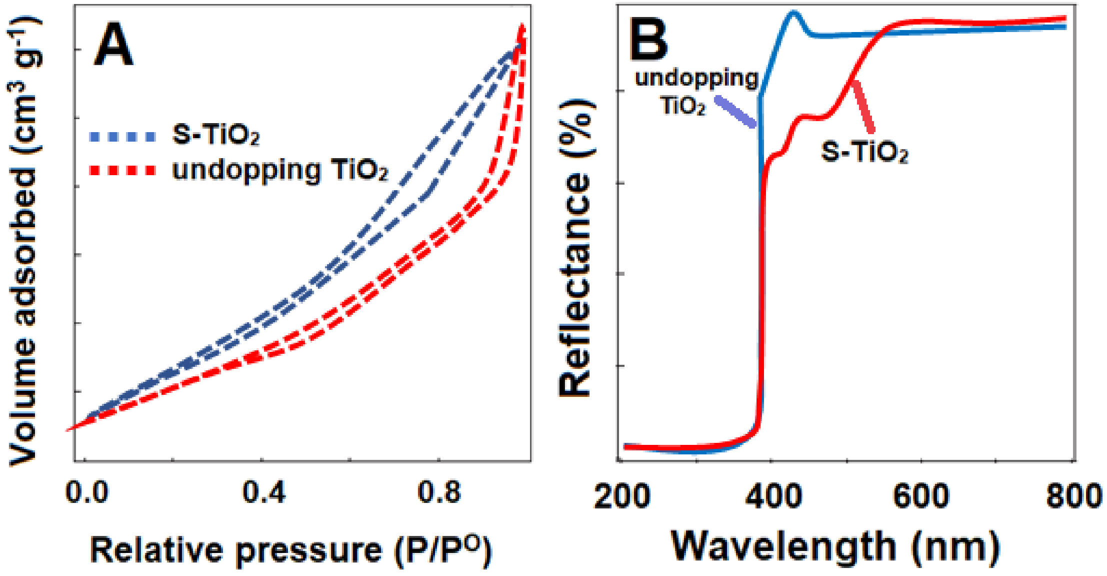

Figure S2. (A) Nitrogen adsorption isotherms of S-TiO_2 and undopping TiO_2 nanomaterials and (B) diffuse reflectance spectra of S-TiO_2 and undopping TiO_2 nanomaterials

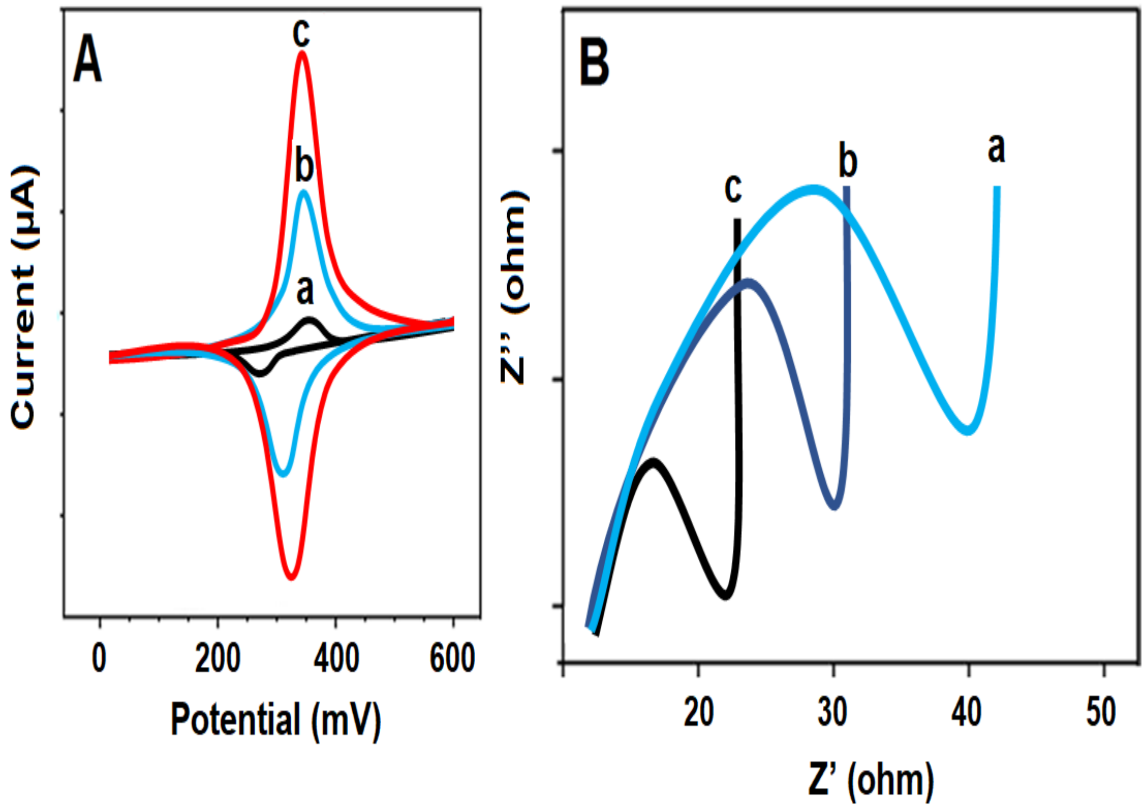

Figure S3. (A) CV curves and (B) EIS responses at (a) bare GCE, (b) undopping TiO_2/GCE, and (c) S-TiO_2/GCE (Redox probe: 5.0 mM $[Fe(CN)_6]^{3-/4-}$ containing 0.1 M KCl, potential scan rate: 100 mV s^{-1})

Table S1. Recovery results of THI (n=6)

Sample	Added THI (nM)	Found THI (nM)	*Recovery (%)
Milk	-	0.13 ± 0.06	-
	2.00	2.14 ± 0.05	100.47 ± 0.07
	4.00	4.12 ± 0.07	99.76 ± 0.06
	6.00	6.15 ± 0.01	100.33 ± 0.04

*Recovery = Found THI, nM / Real THI, nM

Table S2. k and k′ values of THI imprinted SPR chips (MIP/S-TiO_2/SPR chip and NIP/S-TiO_2/SPR chip) (n=6)

	MIP		NIP		
	ΔR	k	ΔR	k	k′
THI	5.0 ± 0.03	-	0.10 ± 0.02	-	-
ZIR	0.50 ± 0.01	10.00	0.08 ± 0.04	1.25	8.00
THP	0.40 ± 0.04	12.50	0.06 ± 0.03	1.67	7.44
FER	0.30 ± 0.03	16.67	0.04 ± 0.07	2.50	6.67
DIS	0.20 ± 0.06	25.00	0.02 ± 0.04	5.00	5.00

Analyte concentrations: 10.0 nM THI, 1000.0 nM ZIR, 1000.0 nM THP, 1000.0 nM FER, 1000.0 nM DIS
k (selectivity coefficient) = $\Delta R_{THI}/\Delta R_{interfering\ chemical}$ and k′ (relative selectivity coefficient) = k_{MIP}/k_{NIP}.
